# Malnutrition risk and nutritional interventions among older hospitalised patients with and without dementia: a multicentre nine-year study

**DOI:** 10.1016/j.jnha.2026.101007

**Published:** 2026-10-16

**Authors:** Doris Eglseer, Silvia Bauer

**Affiliations:** Medical University of Graz, Institute of Nursing Science, Graz, Austria

**Keywords:** Dementia, Malnutrition, Hospitalisation, Nutritional interventions, Older adults, Care dependency

## Abstract

**Objectives:**

To (1) describe the prevalence of malnutrition risk in older hospitalised patients with and without dementia across annual cross-sectional assessments between 2017 and 2025; (2) examine nutritional interventions among older hospitalised patients at malnutrition risk with and without dementia between 2017 and 2025; and (3) identify clinical and demographic factors associated with the co-occurrence of dementia and malnutrition risk.

**Design:**

Multicentre, cross-sectional secondary data analysis.

**Setting:**

Austrian acute care hospitals participating in the Nursing Quality Measurement 2.0 (LPZ) between 2017 and 2025.

**Participants:**

A total of 9,991 hospitalised patients aged ≥65 years were included; 616 (6.2%) had a documented dementia diagnosis, 2,369 (23.7%) were at risk of malnutrition, and 144 (1.4%) had both dementia and malnutrition risk.

**Measurements:**

Data collection included a standardized questionnaire using patient interviews, clinical assessments, and review of medical records. Malnutrition risk was assessed using the Malnutrition Universal Screening Tool (MUST). Dementia diagnoses were based on ICD-10 documentation. Thirteen nutritional interventions were recorded dichotomously. Group comparisons and multivariable logistic regression analyses (with co-occurrence of dementia and malnutrition risk according to MUST as the dependent variable) were performed.

**Results:**

The overall malnutrition risk was 23.7% and did not differ between patients with and without dementia (23.4% vs. 23.7%,  =  0.840). Among patients at malnutrition risk, those with dementia more frequently received mealtime assistance (59.7% vs. 15.3%, p < 0.001), modification of the mealtime environment (57.6% vs. 26.3%, p < 0.001), texture-modified diets (47.9% vs. 16.7%, p < 0.001), enriched diets (47.5% vs. 16.0%, p < 0.001) and oral nutritional supplements (28.5% vs. 19.2%, p = .008) than those without dementia. Between 2017 and 2025, the use of several nutritional interventions varied. The co-occurrence of dementia and malnutrition risk was independently associated with female sex (OR 1.56, CI 1.07–2.29), higher age (OR 1.07 per year, CI 1.04–1.09), end-of-life condition (OR 1.89, CI 1.21–2.95), higher number of medical diagnoses (OR 1.37, CI 1.24–1.50), and greater care dependency (OR 0.95 per CDS point, CI 0.94−0.96) in the multivariable analysis.

**Conclusion:**

Older hospitalised patients with dementia and malnutrition risk received a broad range of nutritional interventions in routine clinical practice, particularly interventions supporting oral intake. Nevertheless, variation in nutritional care and the comparatively modest use of some guideline-recommended interventions indicate a need for further research on the appropriateness and implementation of nutritional care in this population.

## 1 Introduction

As society ages, dementia has emerged as one of the most significant diseases preventing healthy aging [1]. Currently, more than 57 million people live with dementia globally, with approximately 10 million new cases being diagnosed each year [2]. This number is projected to increase to nearly 78 million by 2030 [1]. Dementia is a leading cause of care dependency among older adults. Care dependency refers to the extent to which a person requires professional support to meet basic physical, psychological, and social needs, including activities such as eating and drinking, mobility, hygiene, and communication [3]. It is also a major cause of disability and the seventh leading cause of death worldwide [1]. Research has attributed the development of dementia to at least 12 modifiable risk factors—including diet-related factors—such as mid-life obesity, diabetes, smoking, or physical inactivity, suggesting that a substantial proportion of dementia cases may be preventable [4].

Diet-related factors are not only a risk factor for dementia, nutritional problems are also a likely consequence of dementia. Dementia is associated with a wide range of nutrition-related physiological, psychological, and social changes that negatively affect dietary intake [5,6]. Unintentional weight loss is highly prevalent and often occurs already in the pre-diagnostic phase [7,8]. Dysphagia is common and further complicates eating and drinking in later stages of the disease [9]. In addition, psychological and social challenges, such as those associated with shopping, food storage, meal preparation, and eating, can further influence food intake. Behavioural symptoms, including agitation and hyperactivity, can additionally increase energy requirements [5].

Taken together, these factors substantially increase the risk for malnutrition. Malnutrition occurs in all stages of dementia, even in preclinical stages, and becomes more prevalent as the disease severity increases [10]. The prevalence of malnutrition among people with dementia is approximately 33%, depending on the tool/definition used [11]. Malnutrition itself is associated with adverse outcomes, contributes to disease progression, and increases the caregiver burden [15]. Poor nutritional status in people with dementia has been linked to more severe neuropsychiatric symptoms, including depression or apathy [16], as well as to increased morbidity and mortality [17,18].

Hospitalisation represents a critical and vulnerable period for individuals with dementia [19]. This period is associated with an increased risk of adverse outcomes such as delirium or of falls [20], but also with a higher risk of nutritional deterioration [[21], [22], [23]]. Consequently, appropriate nutritional management is a core component of comprehensive dementia care in hospitals. Current guidelines, such as the ESPEN guideline on nutrition and hydration in dementia, outline key nutritional interventions for this population [15].

Nutritional interventions should meet individual needs insofar as possible, and individual dietary counselling by trained dietitians should be offered not only to the patients but also their caregivers [15]. Recommended interventions include offering fortified foods and drinks to increase energy, as well as protein intake and texture-modified foods and drinks if indicated. The only Grade A recommendation is the use of oral nutritional supplements (ONS) to improve energy and protein intake and to maintain or improve nutritional status [15,24,25]. Enteral and parenteral nutrition as well as hydration may be appropriate for a limited period in selected situations; however, these interventions are generally not recommended in the terminal phase of the illness. Decisions on medical nutrition and hydration should always be guided by the individual clinical context and the expressed wishes of the affected person [15,45].

Previous studies have mainly focused on the prevalence of malnutrition risk in people with dementia, often in long-term care settings or small clinical samples, and despite of guidelines that provide recommendations for nutritional management in dementia. Evidence on the type and frequency of nutritional interventions implemented in routine hospital care for patients with dementia remains limited [11,15]. Furthermore, we could not identify studies that examined how nutritional interventions vary across repeated annual measurements. Previous research in older populations has identified several factors associated with malnutrition or malnutrition risk, including older age, functional and eating dependency, frailty, multimorbidity, cognitive impairment, and dementia [12]. However, evidence on the demographic and clinical characteristics associated specifically with the co-occurrence of dementia and malnutrition risk among older hospitalised patients is scarce. Identifying such characteristics may help to recognise particularly vulnerable patient groups requiring comprehensive nutritional assessment and care, which should be based on individual nutritional assessment.

The aims of this study are to (1) describe the *prevalence of malnutrition risk* in older hospitalised patients with and without dementia across annual cross-sectional assessments between 2017 and 2025; (2) examine *nutritional interventions* among older hospitalised patients at malnutrition risk with and without dementia between 2017 and 2025; and (3) identify *clinical and demographic factors* associated with the co-occurrence of dementia and malnutrition risk.

## 2 Material and methods

### 2.1 Study design

This study is a secondary analysis of data from the Nursing Quality Measurement 2.0, also known as the LPZ (*Landelijke Prevalentiemeting Zorgkwaliteit*). The LPZ is an internationally established, multicentre, cross-sectional quality measurement conducted in inpatient and outpatient care settings in various countries. The study targets established nursing care quality indicators, including pressure injuries, incontinence, malnutrition risk, falls, use of restraints, and pain [26]. Data collection takes place once per year during a predefined period in November. In Austria, all hospitals with more than 50 inpatient beds are eligible and invited to participate. For the present analysis, data from hospitalised patients in Austria collected from 2017 to 2025 were used. No data were available for the year 2020, as the study was not conducted that year due to the COVID-19 pandemic.

### 2.2 Ethical considerations

Ethical approval was granted for this study by the responsible ethical committee (Ethics Committee of the Medical University of Graz, EK Nr. 20-192 ex 08/09). Written informed consent (2017–2020) and oral informed consent (2021–2025) was obtained from all participating patients or their legal representatives. Ethical principles for research with humans were adhered to [27].

### 2.3 Data collection

Data were collected on a predefined measurement day. All patients present on the participating wards on that day were eligible for participation, irrespective of their admission date or length of hospital stay before the measurement. Data were collected using a standardised questionnaire. This questionnaire, developed by an interdisciplinary expert panel based on current evidence and expert consensus, has demonstrated good face validity and inter-rater reliability [26]. The data collection was carried out by pairs of trained nurses. One nurse was directly involved in the patient’s care, while the second nurse was independent of the ward, a procedure intended to enhance objectivity and data quality. Data were gathered through patient interviews where possible, clinical assessments, and the systematic review of medical records. All data collectors participated in standardised group training sessions and received written instructions prior to the data collection [26]. The questionnaire was used to assess the organisational information such as the medical specialty and information about the demographic characteristics age and sex, and level of care dependency of participants, as assessed using the Care Dependency Scale (CDS) [3]. The CDS is a 15-item instrument scored on a five-point Likert scale, with lower values indicating greater dependency. CDS categories were derived as follows: 15–24 points indicated complete dependency, 25–44 points mostly dependent, 45–59 points partially dependent, 60–69 points mostly independent, and 70–75 points completely independent. Care dependency was assessed by the trained nurses based on patient observation and, where possible, information provided by the patient.

All diagnoses, including the diagnosis of dementia, were identified from the medical records based on documented medical diagnoses at admission according to the International Classification of Diseases, 10th Revision (ICD-10). In addition, BMI was calculated based on clinically assessed weight measured in kilograms while wearing light clothing and height in centimeters. Information on surgery within the last two weeks (yes/no) and whether a patient was on an end-of-life care pathway (yes/no) was obtained from the medical records. The presence of selected care indicators, including pressure injuries, urinary incontinence, falls, and pain was assessed using the standardized LPZ questionnaire by clinical assessment (pressure injuries, incontinence, pain) and patient interview and medical records (fall). The risk of malnutrition was assessed using the Malnutrition Universal Screening Tool (MUST), a validated and widely used screening instrument in adults across all healthcare settings [28,29]. As MUST is a screening rather than a diagnostic tool, only malnutrition risk could be identified. Actual malnutrition could not be determined because no comprehensive nutritional assessment or Global Leadership Initiative on Malnutrition (GLIM) criteria were available. MUST incorporates Body Mass Index (BMI) (0, 1 or 2 points), unintentional weight loss (0, 1 or 2 points), and the presence of acute disease with no nutritional intake for more than five days (0 or 2 points). Information on unintentional weight loss and nutritional intake was obtained through patient interviews and supplemented by information available to the nurses and in the medical records. Higher scores indicate higher malnutrition risk. These components are summed to generate an overall score, with higher scores indicating a greater risk of malnutrition. Based on the total MUST score, patients were classified as being at low (score 0), medium (score 1), or high (score ≥2) risk of malnutrition. For the present analyses, medium and high risk were combined and classified as malnutrition risk (MUST score ≥1) [29].

In addition, information on nutritional interventions was collected with the standardised questionnaire from patient documentation and information provided by the nurse directly involved in the patient’s care. The use of nutritional interventions included dietitian consultation, provision of ONS, administration of enteral or parenteral nutrition, assistance during mealtimes, modification to the mealtime environment, texture-modified diet, energy/protein enriched diet, information for patients and relatives, energy and/or protein enriched snacks, monitoring nutritional intake, fluid intake monitoring, other interventions. These interventions were recorded using dichotomous (yes/no) response options [26].

### 2.4 Statistical analyses

Descriptive statistics were first used to characterise the study population and variables of interest. Categorical variables are presented as absolute and relative frequencies. Continuous variables are reported as means with standard deviations or as medians with interquartile ranges, depending on data distribution. Group comparisons between patients with and without dementia diagnosis were performed using independent-samples *t*-tests for normally distributed continuous variables, Mann-Whitney *U* tests for non-normally distributed continuous variables, and chi-squared tests for categorial variables. Differences in the frequency of nutritional interventions between patients with and without dementia were assessed using chi-squared tests. The choice of the appropriate statistical test was based on the scale of measurement and the distribution characteristics of the variables. A heat map was created where lighter colours indicate a higher frequency of interventions.

Comparison of malnutrition prevalence and nutritional interventions between the years of measurement was conducted descriptively. To identify clinical and demographic factors associated with the co-occurrence of malnutrition risk and dementia, univariable and multivariable binary logistic regression analyses using the enter method were conducted. The dependent variable was the co-occurrence of dementia and malnutrition risk (yes/no). Patients with both conditions (n = 144) were compared with all other patients in the sample (n = 9,847; total N = 9,991). The specific clinical and demographic covariates were selected *a priori* based on evidence and clinical relevance, including sex, age in years, BMI, surgery within the last two weeks, end-of-life condition, cardiovascular disease, disease of the musculoskeletal system, disease of the genitourinary system, endocrine/nutritional/metabolic disease, disease of the respiratory system, diabetes mellitus, care dependency score, care dependency in eating/drinking, pressure ulcer, urinary incontinence, number of medical diagnoses, falls during hospitalization within the previous 30 days, and daily pain in the last seven days.

Variables showing an association with the outcome co-occurrence of dementia and malnutrition risk at a *p*-value < 0.30 in univariable analyses were entered into the multivariable model. Prior to performing the multivariable analysis, the multicollinearity among the independent variables was assessed using the variance inflation factor (VIF); values below three were considered indicative of acceptable collinearity [30]. The model fit was evaluated using the Omnibus Tests of Model Coefficients. A *p*-value < 0.05 in the Omnibus test was interpreted as evidence of a statistically significant model. The proportion of explained variance was assessed using Cox–Snell *R*^2^ and Nagelkerke’s *R*^2^. For all statistical analyses, a two-sided *p*-value < 0.05 was considered statistically significant. The adequacy of the multivariable regression model was critically considered in relation to the sample size and number of covariates included.

To handle missing data, a complete-case analysis approach was applied. For the secondary data analysis in this study, we excluded data from persons who were not hospitalised, who refused participation, patients younger than 65 years, as well as patients with missing data on malnutrition risk according to MUST, because malnutrition risk was a main study variable.

## 3 Results

In the years 2017–2025, a total of 32,336 persons were included in the LPZ measurement in Austria. Across the study period, there were 209 hospital participations and 1,164 ward participations across the annual measurement years, with some hospitals and wards participating in multiple years. The final sample for this study included 9,991 (*N*) hospitalised patients aged 65 years or older with complete data on malnutrition risk. Fig. 1 shows the participant selection process.

**Fig. 1:**
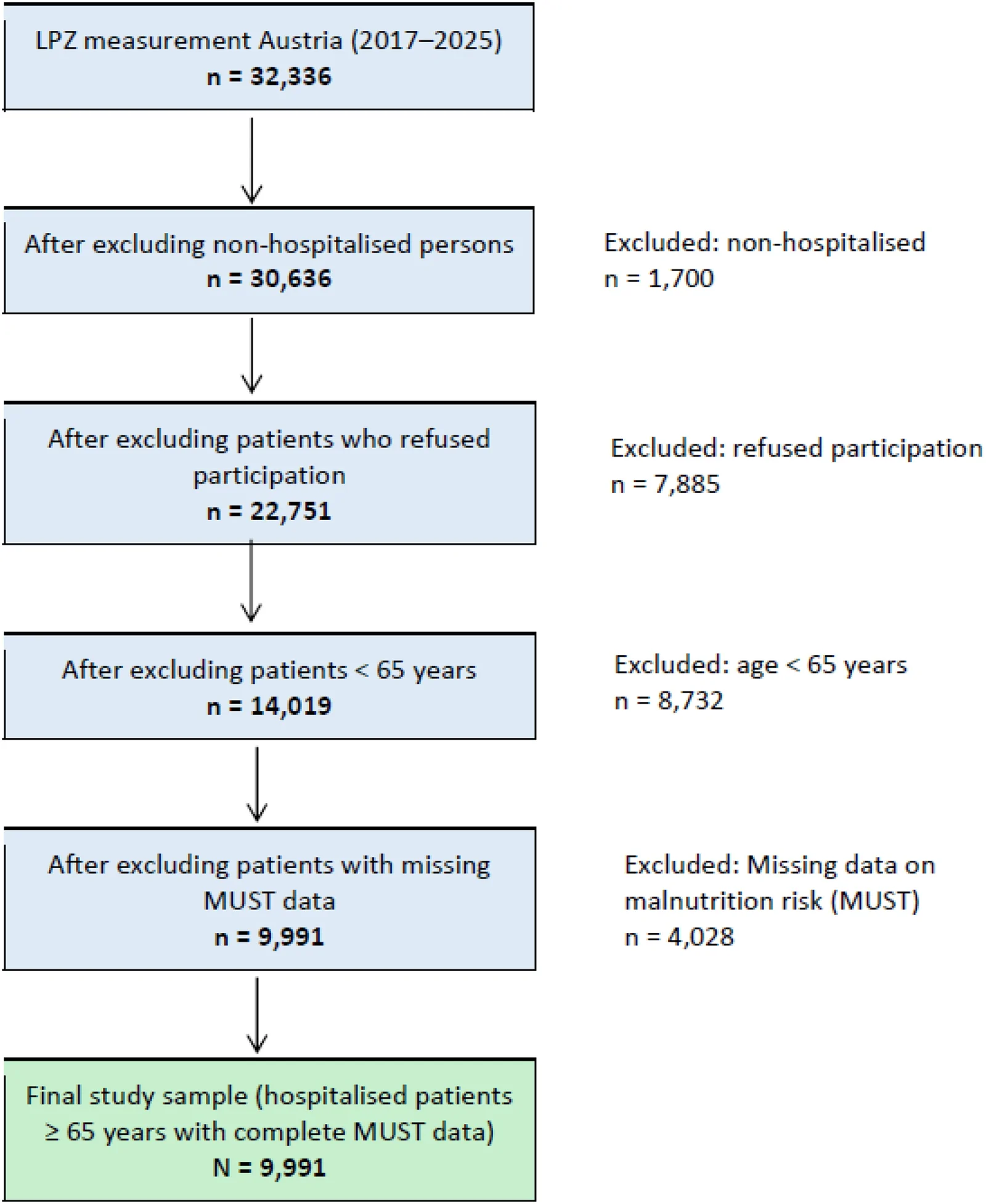
Flowchart of participant selection for the present study. LPZ = Landelijke Prevalentiemeting Zorgproblemen; MUST = Malnutrition Universal Screening Tool.

### 3.1 Patient characteristics

Mean age of the study sample was 77.8 years, 53.4% were female, median number of medical diagnoses was three and the prevalence of malnutrition risk was 23.7%. The prevalence of dementia was 6.2% (*n* = 616). Dementia prevalence was higher among excluded participants with missing MUST scores (9.9%) than among those with malnutrition risk (6.1%) or those without malnutrition risk according to MUST (6.2%).

Table 1 shows the characteristics of the total study sample, as well as those of patients with and without dementia. Compared with patients without dementia, patients with dementia were older, more often female, had lower BMI, a higher number of medical diagnoses, greater care dependency, and a higher prevalence of end-of-life conditions, pressure injuries, urinary incontinence, and falls during hospitalisation within 30 days. They were also more frequently treated in geriatric and psychiatric wards and had less frequently undergone surgery within the previous two weeks. No association was found between dementia and malnutrition risk (*p* = 0.840).

**Table 1:** Patient characteristics for the entire study sample and according to an existing dementia diagnosis.

	Total (N = 9991)	Patients with dementia (*n* = 616)	Patients without dementia (*n* = 9375)	*p*-value *
Female sex, *n* (%)	5331 (53.4)	390 (63.3)	4941 (52.7)	< 0.001
Age in years, mean (SD)	77.8 (7.7)	83.7 (7.2)	77.4 (7.6)	< 0.001
BMI in kg/m^2^, mean (SD)	26.3 (5.3)	24.2 (4.6)	26.4 (5.3)	< 0.001
Number of medical diagnoses, median (IQR)	3 (2−4)	4 (3−6)	3 (2−4)	< 0.001
Specialty of patients/ward				< 0.001
Surgical ward	3014 (30.2)	75 (12.2)	2939 (31.3)	
Medical ward	5348 (53.5)	291 (47.2)	5057 (53.9)	
Geriatric ward	1005 (10.0)	191 (31.0)	814 (8.6)	
Psychiatric ward	174 (1.7)	29 (4.7)	149 (1.5)	
Others	450 (4.6)	30 (4.9)	416 (4.7)	
Surgery in the last 2 weeks, *n* (%)	2220 (22.2)	52 (8.4)	2168 (23.1)	< 0.001
End-of-life condition, *n* (%)	571 (5.7)	80 (13.0)	491 (5.2)	< 0.001
Most frequent diagnoses				
Cardiovascular disease	5944 (59.5)	450 (73.1)	5494 (58.6)	< 0.001
Disease of the musculoskeletal system	2808 (28.1)	200 (32.5)	2608 (27.8)	0.013
Disease of the genitourinary system	2487 (24.9)	219 (35.6)	2268 (24.2)	< 0.001
Endocrine/nutritional/metabolic disease	2382 (23.8)	178 (28.9)	2205 (23.5)	0.002
Disease of the respiratory system	2314 (23.2)	118 (19.2)	2196 (23.4)	0.015
Diabetes mellitus	2192 (21.9)	154 (25.0)	2038 (21.7)	0.058
Care dependency score, mean (SD)	63.8 (14.7)	46.2 (16.5)	65.0 (13.8)	< 0.001
Care dependency, according to the CDS				< 0.001
Completely dependent	345 (3.5)	82 (13.3)	263 (2.8)	
Mostly dependent	834 (8.3)	194 (31.5)	640 (6.8)	
Partially dependent	1547 (15.5)	188 (30.5)	1359 (14.5)	
Mostly independent	2024 (20.3)	99 (16.1)	1925 (20.5)	
Completely independent	5241 (52.4)	53 (8.6)	5188 (55.4)	
Care dependency in eating and drinking, according to the CDS	2205 (22.1)	355 (57.6)	1850 (19.7)	< 0.001
Additional nursing care indicators				
Pressure injury	366 (3.7)	42 (6.9)	324 (3.5)	< 0.001
Urinary incontinence	2376 (24.1)	353 (57.6)	2023 (21.9)	< 0.001
Fall during the hospital stay within the last 30 days	507 (5.1)	67 (10.9)	440 (4.7)	< 0.001
Pain in the last 7 days				< 0.001
Yes, but not on a daily basis	2474 (25.4)	144 (23.4)	2330 (24.9)	
Daily pain	2912 (29.9)	102 (16.6)	2810 (30.0)	
Year of participation, *n* (%)				< 0.001
2017	1670 (16.7)	104 (16.9)	1566 (16.7)	
2018	1736 (17.4)	80 (13.0)	1656 (17.7)	
2019	1482 (14.8)	115 (18.7)	1367 (14.6)	
2021	476 (4.8)	32 (5.2)	444 (4.7)	
2022	1427 (14.3)	66 (10.7)	1361 (14.5)	
2023	1203 (12.0)	79 (12.8)	1124 (12.0)	
2024	744 (7.4)	61 (9.9)	683 (7.3)	
2025	1253 (12.5)	79 (12.8)	1174 (12.5)	

CDS = Care Dependency Scale.

* Analysed with the Mann–Whitney *U* test or the chi-squared test depending on the data.

### 3.2 Malnutrition risk prevalence

The overall prevalence of malnutrition risk according to the MUST was 23.7% for the total sample and 23.4% for patients with dementia based on data from all years combined. As shown in Table 2, patients with dementia more frequently had a BMI below 20 kg/m2, but less frequently experienced unintentional weight loss of ≥5% or acute illness with reduced nutritional intake than patients without dementia. Across the study years, the prevalence of malnutrition risk varied from 16.3% to 33.3% among patients with dementia and from 18.2% to 27.2% among patients without dementia (Fig. 2).

**Table 2:** Malnutrition risk according to MUST.

	Total (N = 9991)	Patients with dementia (*n* = 616)	Patients without dementia (*n* = 9375)	*p*-value
Malnutrition risk (MUST) total, *n* (%)*	2369 (23.7)	144 (23.4)	2225 (23.7)	0.84
Low risk	7622 (76.3)	472 (76.6)	7150 (76.3)	
Medium risk	430 (4.3)	35 (5.7)	395 (4.2)	
High risk	1939 (19.4)	109 (17.7)	1830 (19.5)	
BMI				< 0.001
>20 kg/m^2^	9099 (91.1)	510 (82.8)	8589 (91.6)	
18.5−20 kg/m^2^	479 (4.8)	50 (8.1)	429 (4.6)	
<18.5 kg/m^2^	413 (4.1)	56 (9.1)	357 (3.8)	
Unplanned weight loss last 3−6 months				0.01
<5%	9043 (90.5)	578 (93.8)	8465 (90.3)	
5−10%	269 (2.7)	8 (1.3)	261 (2.8)	
>10%	679 (6.8)	30 (4.9)	649 (6.9)	
Acute illness and reduced/no intake	1240 (12.4)	42 (6.8)	1198 (12.8)	<0.001

* MUST includes BMI, unintentional weight loss, and acute disease with reduced/no nutritional intake for >5 days. Patients with a MUST score of 0 were classified as not at risk of malnutrition, and those with a score ≥1 as at risk.

**Fig. 2:**
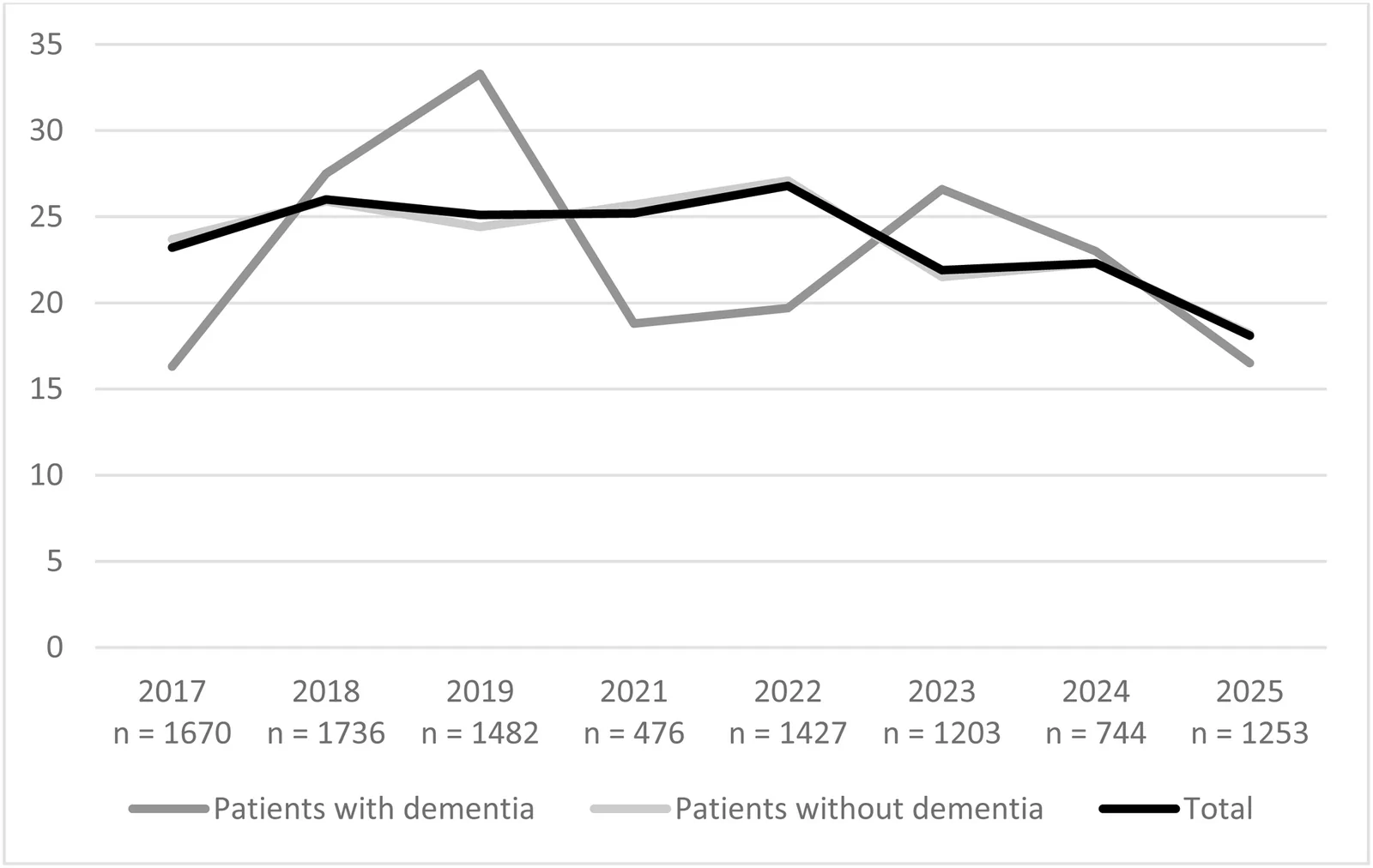
Prevalence of malnutrition risk for patients with and without dementia in the study period.

### 3.3 Nutritional interventions among patients at risk of malnutrition

Among patients at risk of malnutrition, 89% received at least one nutritional intervention. This proportion was higher among patients with dementia, of whom 96% received at least one nutritional intervention. As shown in Fig. 3, most nutritional interventions were significantly more frequently applied among patients with dementia than among those without dementia. No significant differences were observed for dietitian consultation, enteral nutrition, or parenteral nutrition.

**Fig. 3:**
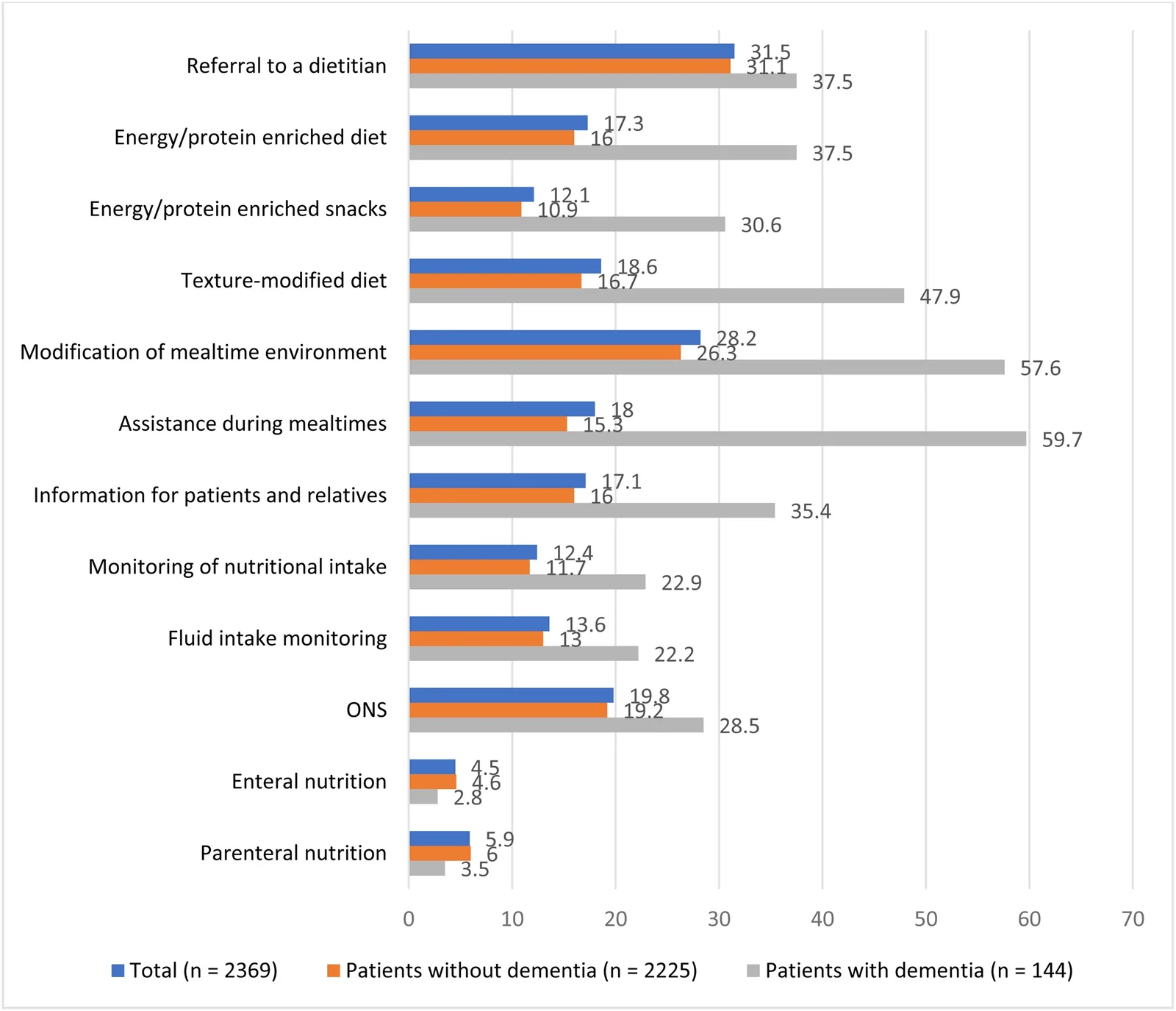
Nutritional interventions in hospitalised older patients at malnutrition risk, with and without dementia.

* The frequency of interventions differed significantly between patients at risk of malnutrition with and without dementia, except for the following interventions: enteral nutrition, parenteral nutrition, referral to a dietitian, other interventions

Across the repeated annual cross-sectional assessments, the frequency of nutritional interventions varied considerably among patients with dementia and malnutrition risk (Fig. 4). The greatest variability was observed for energy- and protein-enriched diets and texture-modified diets, both of which peaked in 2021 and were applied less frequently in subsequent years. In contrast, the use of enteral and parenteral nutrition remained consistently low throughout the study period.

**Fig. 4:**
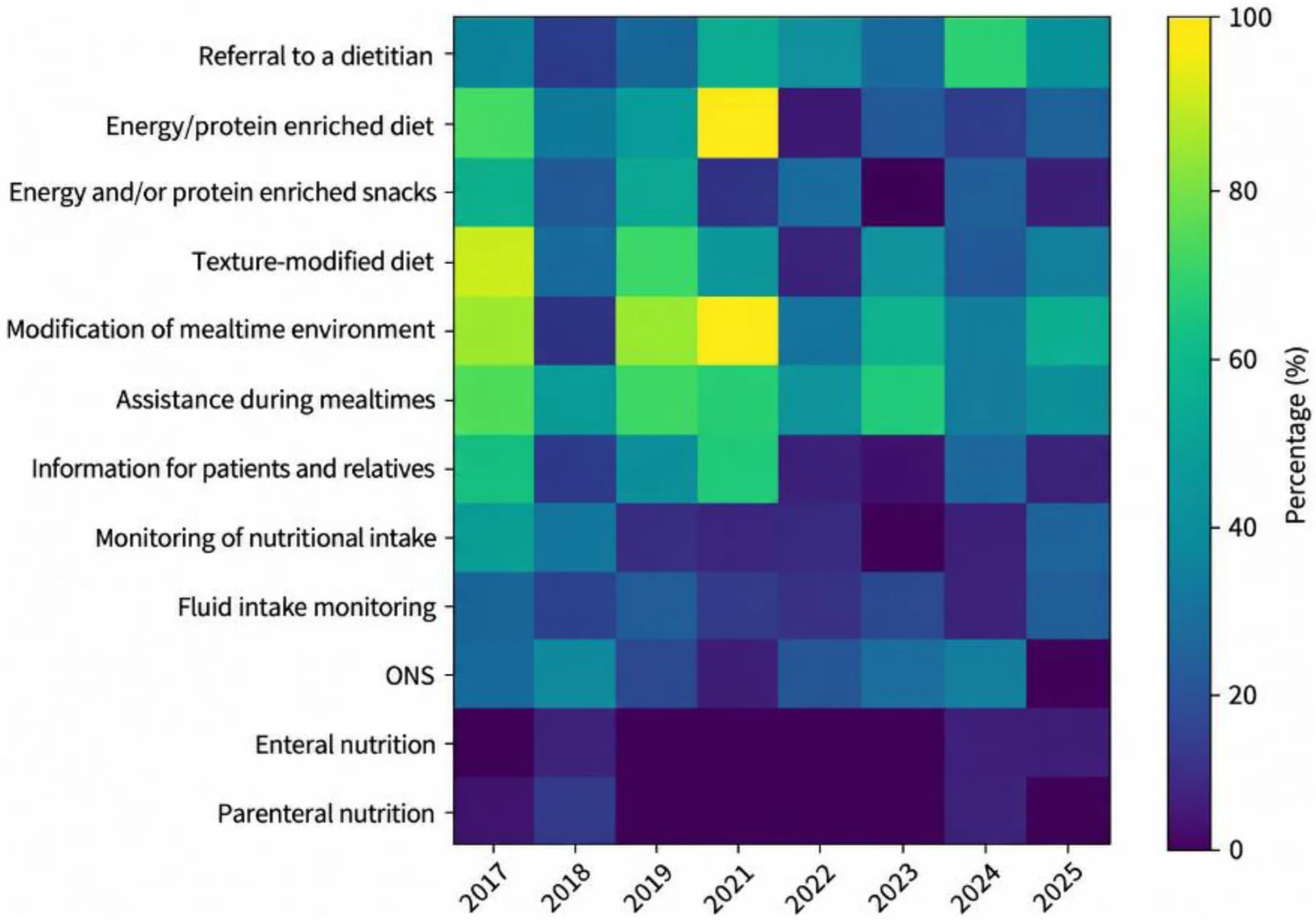
Heat map with percentage of patients receiving each intervention in each measurement year for patients with dementia and malnutrition risk in the study period (n = 144 for all years); lighter shading indicates higher intervention frequency. The number of patients participating in each study year is reported in Table 1; the present figure includes only patients with dementia and malnutrition risk.

### 3.4 Factors associated with the co-occurrence of dementia and malnutrition risk

A total of 144 participants (1.4%) had both dementia and malnutrition risk. In the univariable regression analyses, all investigated variables except year of participation, endocrine/nutritional/metabolic disease, respiratory disease, and diabetes mellitus were significantly associated with the co-occurrence of dementia and malnutrition risk (Table 3)

**Table 3:** Univariable and multivariable logistic regression results (dependent variable: diagnosis dementia combined with malnutrition risk according to MUST, n = 144).

Variable	*B*	SE	Univariable OR (95% CI)	*p*-value	*B*	SE	Multivariable OR (95% CI)	*p*-value
Year of participation	−0.01	0.03	0.99 (0.94–1.05)	0.85				
Sex (ref: male)	0.66	0.18	1.94 (1.36–2.77)	< 0.001	0.45	0.19	1.56 (1.07–2.29)	0.02
Age in years	0.10	0.01	1.10 (1.09–1.13)	< 0.001	0.07	0.01	1.07 (1.04–1.09)	< 0.001
Surgery within the last 2 weeks	−1.16	0.30	0.32 (0.17–0.57)	< 0.001	−0.54	0.32	0.58 (0.31–1.08)	0.09
End-of-life condition	1.64	0.20	5.14 (3.45–7.66)	< 0.001	0.63	0.23	1.89 (1.21–2.95)	0.005
Cardiovascular disease	0.69	0.19	1.99 (1.36–2.89)	< 0.001	−0.09	0.22	0.92 (0.59–1.42)	0.70
Disease of the musculoskeletal system	0.41	0.17	1.50 (1.07–2.11)	0.02	−0.19	0.21	0.83 (0.55–1.24)	0.36
Disease of the genitourinary system	0.69	0.172	2.0 (1.43–2.80)	< 0.001	−0.08	0.20	0.92 (0.62–1.37)	0.69
Endocrine/nut-ritional/meta-bolic disease	0.17	0.19	1.19 (0.82–1.72)	0.36				
Disease of the respiratory system	−0.05	0.20	0.95 (0.64–1.41)	0.79				
Diabetes mellitus	−0.20	0.22	0.82 (0.54–1.25)	0.35				
Care dependency score	−0.06	0.00	0.94 (0.93–0.95)	< 0.001	−0.05	0.01	0.95 (0.94–0.96)	< 0.001
Care dependency in eating/drinking	1.87	0.18	6.5 (4.59–9.13)	< 0.001	−0.12	0.28	0.89 (0.52–1.52)	0.66
Pressure ulcer	0.80	0.32	2.22 (1.19–4.15)	0.01	−0.25	0.34	0.78 (0.40–1.52)	0.46
Urinary incontinence	1.45	0.17	4.28 (3.06–5.97)	< 0.001	−0.03	0.21	0.97 (0.65–1.45)	0.88
Number of medical diagnoses	0.37	0.03	1.44 (1.35–1.54)	< 0.001	0.31	0.05	1.37 (1.24–1.50)	< 0.001
Fall within the last 30 days in the hospital	0.71	0.29	2.04 (1.17–3.57)	0.01	0.09	0.31	1.09 (0.60–2.01)	0.77
Daily pain in the last 7 days	−0.66	0.22	0.52 (0.34–-0.80)	0.003	−0.45	0.24	0.64 (0.40–1.01)	0.05

B = Regression Coefficient; SE = Standard Error; OR = Odds Ratio; CI = Confidence Interval.

In the multivariable model, female sex (OR 1.56, 95% CI 1.07–2.29), higher age (OR 1.07 per year, 95% CI 1.04–1.09), end-of-life condition (OR 1.89, 95% CI 1.21–2.95), a higher number of medical diagnoses (OR 1.37, 95% CI 1.24–1.50), and lower care dependency score (meaning higher care dependency) (OR 0.95, 95% CI 0.94–0.96) were independently associated with increased odds of the co-occurrence of dementia and malnutrition risk. The logistic regression model was statistically significant (Omnibus test, χ^2^ (15) = 321.77, p < 0.001) and explained 3.3% of the variance according to Cox–Snell R^2^ and 23.1% according to Nagelkerke R^2^. No relevant multicollinearity was observed among the variables included in the multivariable model (all VIFs < 3). Table 3 displays the results of the univariable and multivariable logistic regression analyses in detail.

## 4 Discussion

This study examined not only malnutrition risk in older hospitalised patients with and without dementia but also the application frequency of nutritional interventions among patients at malnutrition risk with and without dementia across repeated annual cross-sectional assessments. A particular contribution of this study is the description of nutritional interventions documented in routine hospital care among patients with malnutrition risk with and without dementia, an aspect for which evidence has previously been limited. Approximately one in four patients with dementia was classified as being at risk of malnutrition (23.4%), results that are nearly identical for patients without dementia (23.7%). Among patients at malnutrition risk, those with dementia more frequently received nutritional interventions than those without dementia, including environmental adaptations, assistance during mealtimes, texture-modified diets, enrichment of meals and snacks, monitoring of food and fluid intake, provision of nutritional information to patients and relatives, and ONS. Descriptive analyses from 2017 to 2025 revealed considerable variation in the prevalence of malnutrition risk in patients with and without dementia and the frequency of nutritional interventions among patients with malnutrition risk and dementia. The use of enteral and parenteral nutrition remained consistently low in this subgroup over the years. In the multivariable model, female sex, higher age, end-of-life condition, greater care dependency, and a higher number of medical diagnoses were associated with the co-occurrence of dementia and malnutrition risk.

### 4.1 Malnutrition risk prevalence and interventions

The malnutrition risk prevalence did not differ between patients with and without dementia in our sample, although this sample displayed significant differences in age, comorbidity burden, and care dependency. These findings contrast with those of previous studies, where dementia has typically been associated with a substantially higher prevalence of malnutrition and malnutrition risk [[11], [12], [13], [14]], but are consistent with a matched cohort study showing that hospital-acquired malnutrition did not differ in patients with and without dementia [31]. However, the individual MUST components differed between patients with and without dementia. Patients with dementia more frequently had a low BMI, whereas unintentional weight loss and acute illness with reduced intake were less frequently recorded. This highlights the multifactorial nature of malnutrition risk in older hospitalised patients, may partly explain the comparable overall MUST prevalence observed in the two groups and suggests that the choice of screening parameters may influence risk classification.

Screening tools specifically developed for older adults, such as the MNA, which additionally considers factors such as mobility and neuropsychological problems, might therefore have yielded different results. One possible explanation for our findings is that the hospitalisation in older adults is often accompanied by acute illness, multimorbidity, polypharmacy, and other hospital-related barriers to adequate food intake; therefore, they might attenuate dementia-specific effects in the hospital setting [32,33]. Moreover, reliable information on recent weight loss, one of the three components of MUST, may be difficult to obtain in patients with dementia [28,29]. In our data, a substantial number of patients were excluded because of missing MUST data. As dementia was more prevalent among excluded patients with missing MUST data than among patients with available MUST data, this may have introduced selection bias and affected the comparison of malnutrition risk between patients with and without dementia. Another possible explanation for the differences between our findings and those of previous studies is that the awareness of nutritional problems among dementia patients is high among nursing staff as suggested by the higher rate of nutritional interventions.

Although the overall prevalence of malnutrition risk was similar in patients with and without dementia, among patients at malnutrition risk, nutritional interventions were applied substantially more frequently to those with dementia than to those without dementia. This may be due to the greater visibility of several eating-related problems in patients with dementia, including dysphagia, apraxia, attentional deficits, and behavioural symptoms during meals [6,10,34]. These eating-related problems are also reflected in the much higher care dependency in the item eating and drinking (in our sample, 63.9% in patients with dementia vs. 26.4% in patients without dementia). This visible care dependency may motivate nursing staff to provide interventions, such as mealtime assistance or environmental adaptations, simply because they are obvious.

The nutritional interventions conducted in older hospitalised patients with dementia and malnutrition risk mainly attempted to increase oral nutritional intake. Enteral and parenteral nutrition were rarely used. From a clinical perspective, these findings can be interpreted positively: In clinical guidelines, medical nutrition is only recommended as a temporary intervention and only if patients are not in an end-of-life stage [15]. In a terminal stage, providing enteral and parenteral nutrition as well as hydration incur substantial risks and the benefits are uncertain [15]. In comparison, orally fed dementia patients at the end-of-life display a lower prevalence of neuropsychiatric symptoms and higher quality of life [35].

The referral rate to dietitians among patients with dementia and malnutrition risk was relatively low (37.5%), despite recommendations for further nutritional assessment and individualized nutritional care in patients identified as being at nutritional risk [15]. The frequency of specific nutritional interventions, such as ONS, cannot be interpreted in terms of adequacy based on our data, as their use depends on individual nutritional needs, clinical conditions, and treatment goals. In addition, the use frequency of nutritional interventions in this population varied considerably across the measurement years, and especially the frequency of conducting time-intensive measures such as mealtime assistance, the modification of mealtime environment, and the provision of nutritional information to patients and caregivers. Although education on nutritional care is explicitly recommended in the ESPEN guideline on nutrition and hydration in dementia [15], increasing workloads, staff shortages, and time constraints in hospital nursing may limit the delivery of such interventions. Nursing staff shortages are associated with a lack of nursing care and reduced use of malnutrition risk screening, which may negatively affect overall nutritional management [[36], [37], [38]]. Moreover, declining or insufficiently supported positive attitudes toward nutritional care among healthcare professionals may further reduce the implementation of nutritional interventions [39,40]. However, because yearly numbers of patients with both dementia and malnutrition risk were small in our analysis, these descriptive year-specific results should be interpreted cautiously and should not be understood as robust evidence of temporal trends. The observed variation across annual cross-sectional assessments may partly reflect differences in the annually participating hospitals and patient populations, as well as selective participation, rather than changes in nutritional care over time. Furthermore, the present data provide information on whether specific nutritional interventions were documented but do not allow conclusions regarding their appropriateness for individual patients. Future prospective studies should therefore investigate the determinants of intervention use and evaluate whether nutritional interventions are appropriately tailored to individual nutritional needs and clinical conditions.

### 4.2 Factors associated with the co-occurrence of dementia and malnutrition risk

The multivariable regression analysis results reveal that the female sex, older age, end-of-life condition, greater care dependency, and a higher number of medical diagnoses are factors that were associated with the risk of dementia and malnutrition risk. These findings underscore the fact that patients with dementia and malnutrition risk represent a particularly frail and vulnerable patient group [41,42]. Care dependency remained independently associated with the co-occurrence of dementia and malnutrition risk, consistent with previous evidence linking dementia with limitations in activities of daily living [43]. Interestingly, care dependency specifically related to eating and drinking was no longer significant after adjustment, suggesting that global dependency and overall disease burden may be more relevant associated factors for the co-occurrence of dementia and malnutrition risk than isolated deficits. The association between end-of-life condition and malnutrition risk highlights the need for careful goal-oriented nutritional decision-making that balances comfort, burden, and patient preferences [44]. These findings may help draw attention to the nutritional needs of older hospitalised patients with complex clinical and functional care needs, while nutritional management should remain based on individual screening and comprehensive nutritional assessment.

### 4.3 Strengths and limitations

The strengths of this study include its large, multicentre sample, long observation period, and use of standardised data collection procedures. However, several limitations must be considered. The cross-sectional study design does not allow causal inferences to be drawn. The study participation by hospitals and patients was voluntary, which may limit the generalisability of the findings and could have led to the underrepresentation of patients with dementia. Dementia diagnoses were based on medical records and were not independently verified. As diagnostic and documentation practices may vary across hospitals and medical specialties, the recorded diagnoses may not fully reflect the true prevalence of dementia, with a possible underrepresentation of milder or early-stage cases. Information on dementia severity, subtype, and disease stage was not available, although these factors are relevant for nutritional outcomes and nursing care. Furthermore, dementia prevalence was higher among participants with missing MUST scores (9.9%) compared to those with malnutrition risk (6.1%) and those without malnutrition risk according to MUST (6.2%). Consequently, a considerable number of patients with dementia (*n* = 397) were excluded from the analyses due to missing data on nutritional status assessed by MUST. Moreover, the MUST screening tool was not developed specifically for older adults; therefore, it might underestimate malnutrition risk in this population. In addition, acute illness, fluid imbalances, and age-related changes in body composition may limit the sensitivity of BMI-based screening approaches in older hospitalised populations. Furthermore, the subgroup of patients with both dementia and malnutrition risk was relatively small (n = 144), limiting statistical power, increasing uncertainty around the regression estimates, and restricting the number of covariates that could be reliably interpreted. Finally, nutritional interventions were recorded dichotomously, meaning that no information on their intensity, quality, adherence, clinical indication, or appropriateness for individual patients was available.

## 5 Conclusion

The prevalence of malnutrition risk was similar among older hospitalized patients with and without dementia, although differences were observed in the individual MUST components. Among patients at malnutrition risk, those with dementia more frequently received several nutritional interventions than those without dementia. Female sex, older age, end-of-life condition, greater care dependency, and a higher number of medical diagnoses were associated with the co-occurrence of dementia and malnutrition risk. These findings provide insight into differences in nutritional problems and nutritional care among older hospitalized patients with and without dementia. Future prospective studies should investigate the determinants, appropriateness, and effectiveness of nutritional interventions for older hospitalised patients with dementia and malnutrition risk.

## Data

Due to data protection regulations, the datasets analyzed during the current study are not publicly available but are available from the corresponding author upon reasonable request.

## Author contributions

DE: Conceptualisation; Formal analysis; Methodology; Writing – original draft.

SB: Conceptualisation; Methodology; Project administration; Writing – review & editing.

## Declaration of Generative AI and AI-assisted technologies in the writing process

The authors used ChatGPT (OpenAI) to improve English language and readability and to assist in preparing Fig. 4. The authors reviewed and edited all AI-assisted content and take full responsibility for the final manuscript.

## Funding

This research did not receive any specific grant from funding agencies in the public, commercial, or not-for-profit sectors.

## Declaration of competing interest

The authors declare no conflict of interest. SB is the project leader of the Austrian LPZ measurement.
